# A Vaccine Based on Kunitz-Type Molecule Confers Protection Against *Fasciola hepatica* Challenge by Inducing IFN-γ and Antibody Immune Responses Through IL-17A Production

**DOI:** 10.3389/fimmu.2020.02087

**Published:** 2020-10-20

**Authors:** Leonardo Silvane, Daiana Pamela Celias, Pablo Alberto Romagnoli, Belkys Angélica Maletto, María Fernanda Sanchez Vallecillo, Laura Silvina Chiapello, Santiago Daniel Palma, Daniel Alberto Allemandi, Rodrigo Eduardo Fabrizio Sanabria, César Iván Pruzzo, Claudia Cristina Motrán, Laura Cervi

**Affiliations:** ^1^Departamento de Bioquímica Clínica, Facultad de Ciencias Químicas, Universidad Nacional de Córdoba, Córdoba, Argentina; ^2^Centro de investigaciones en Bioquímica Clínica e Inmunología (CIBICI), Consejo Nacional de Investigaciones Científicas y Técnicas (CONICET), Córdoba, Argentina; ^3^Centro de Investigación en Medicina Traslacional Severo Amuchastegui (CIMETSA), Córdoba, Argentina; ^4^Instituto Universitario de Ciencias Biomédicas de Córdoba (IUCBC), Consejo Nacional de Investigaciones Científicas y Técnicas (CONICET), Córdoba, Argentina; ^5^Departamento de Farmacia, Facultad de Ciencias Químicas, Universidad Nacional de Córdoba, Córdoba, Argentina; ^6^Unidad de Investigación y desarrollo en Tecnología Farmacéutica (UNITEFA), Consejo Nacional de Investigaciones Científicas y Técnicas (CONICET), Córdoba, Argentina; ^7^Facultad de Ciencias Veterinarias, Universidad Nacional de La Plata, La Plata, Argentina; ^8^Instituto Tecnológico Chascomús (INTECH), Consejo Nacional de Investigaciones Científicas y Técnicas, Universidad Nacional de San Martín (CONICET/UNSAM), Chascomús, Argentina

**Keywords:** Th17-dependent protection, nanostructure, ascorbyl palmitate, kunitz type molecule, vaccine, *Fasciola hepatica*

## Abstract

*Fasciola hepatica* is helminth parasite found around the world that causes fasciolosis, a chronic disease affecting mainly cattle, sheep, and occasionally humans. Triclabendazole is the drug of choice to treat this parasite. However, the continuous use of this drug has led to the development of parasite resistance and, consequently, the limitation of its effectiveness. Hence, vaccination appears as an attractive option to develop. In this work, we evaluated the potential of *F. hepatica* Kunitz-type molecule (FhKTM) as an antigen formulated with a liquid crystal nanostructure formed by self-assembly of 6-O-ascorbyl palmitate ester (Coa-ASC16) and the synthetic oligodeoxynucleotide containing unmethylated cytosine-guanine motifs (CpG-ODN) during an experimental model of fasciolosis in mice, and we further dissected the immune response associated with host protection. Our results showed that immunization of mice with FhKTM/CpG-ODN/Coa-ASC16 induces protection against *F. hepatica* challenge by preventing liver damage and improving survival after *F. hepatica* infection. FhKTM/CpG-ODN/Coa-ASC16-immunized mice elicited potent IFN-γ and IL-17A with high levels of antigen-specific IgG1, IgG2a, and IgA serum antibodies. Strikingly, IL-17A blockade during infection decreased IgG2a and IgA antibody levels as well as IFN-γ production, leading to an increase in mortality of vaccinated mice. The present study highlights the potential of a new vaccine formulation to improve control and help the eradication of *F. hepatica* infection, with potential applications for natural hosts such as cattle and sheep.

## Introduction

Fasciolosis is a zoonotic and chronic disease caused by a helminth parasite, *F. hepatica*, that causes huge economic losses in animal production worldwide. These losses have been estimated to be US$ 3 billion due to a reduction in milk, wool, and meat production in cows and sheep (1, 2). On the other hand, the World Health Organization (WHO) has reported that approximately 2.4 million people are infected by this parasite worldwide. Fasciolosis has recently been declared as an emerging disease in humans with an increased number of cases in some regions of the planet (3). The frontline drug against fasciolosis is triclabendazole. However, the emergence of resistance to this drug in diverse *F. hepatica* populations (4, 5), and its high cost suggest the need for other control strategies. In this sense, developing a vaccine against this parasite would be a better preventive control strategy. Moreover, vaccines are considered safe and environmentally friendly because their use ensures the absence of chemical residues in food, as well as in pasture (1).

Both cattle and humans are infected by the ingestion of metacercariae, the infective stage, encysted in aquatic plants. After that, the newly excysted juveniles (NEJ) fluke emerges in the intestine and penetrates the intestinal wall in its migration through the peritoneum and liver parenchyma to finally allocate in the bile ducts.

During its migration, the parasite releases an array of molecules from its intestinal content called excretory-secretory products (FhES) or its tegumental coat (FhTeg), which are the main source of immune-modulatory molecules (6–8).

These complex parasite-derived molecules can interact with the immune system and inhibit the Th1-driven protective pro-inflammatory responses through the induction of M2 macrophages (9), mast cells (10), and Th2-type responses (11, 12), and promote regulatory T (Treg) cell development (6, 13). Thus, the induction of an ineffective immune response against this parasite allows the development of a chronic infection.

Over the last 25 years, there have been numerous attempts to formulate a successful vaccine against *F. hepatica* by using parasite extracts or individual antigens (14, 15). These formulations achieved different levels of protection in experimental models of mice, rats, sheep, and cattle (1, 16, 17) by the induction of an antibody response and/or Th1/Th17-mediated cellular immunity (18–22).

However, a commercially viable vaccine against *F. hepatica* with an appropriate level of efficacy is not available yet.

Among the molecules released by the parasite, the most abundant are proteases and protease inhibitors (14, 23, 24). The proteases secreted by *F. hepatica* allow its migration through the tissues and modulate the immune system, which enables its establishment and permanence in the host. However, a tight control of this enzymatic activity should be regulated by protease inhibitors. Kunitz type molecule (FhKTM) is a member of the inhibitory protease family expressed in the FhES and FhTeg during the juvenile stage, suggesting an essential role in controlling proteolytic activity (25). Thus, the physiological function of FhKTM may be to protect the parasite from the host and parasite proteases by inhibiting its activity. In our study we tested a FhKTM peptide as a vaccine antigen.

On the other hand, new approaches have focused on the design of innovative methods to improve immune response involving mainly suitable adjuvant strategies (26). Over the last decade, an area of extreme development has been the application of nanomaterials to vaccine development. In this line, the adjuvant capacity of the synthetic oligodeoxynucleotide containing unmethylated cytosine-guanine motifs (CpG-ODN) (agonist of TLR9) formulated with liquid crystal-type nanostructures formed by self-assembly from ascorbyl 6-O-palmitate ester (Coa-ASC16) has been demonstrated. The immunization of mice with the ovalbumin (OVA) protein, together with the adjuvant CpG-ODN/Coa-ASC16, induced a potent antigen-specific antibodies and Th1/Th17/CD8 + T-cell cellular responses without toxic systemic effects (27, 28).

In this work, we evaluated the potential of an FhKTM peptide formulated in a nanostructure based on CpG-ODN/Coa-ASC16 as a vaccine during an experimental model of fasciolosis in mice and we further dissected the immune response associated with host protection.

## Materials and Methods

### Animals

Wild-type 8- to 10-week-old female BALB/c mice were obtained from the Faculty of Veterinary Sciences, National University of Litoral (UNL, Argentina) and housed in the Animal Facility of the Faculty of Chemical Sciences, National University of Córdoba.

### Ethics Statement

All animal experiments were approved by and conducted in accordance with the guidelines of the committee for Animal Care and Use of the Faculty of Chemical Sciences, National University of Córdoba (Approval Number HCD 881) in strict accordance with the recommendation of the Guide to the Care and Use of Experimental Animals published by the Canadian Council on Animal Care (OLAW Assurance number A5802-01).

### Antigens and Adjuvant

A FhKTM peptide according to the sequence described by Bozas et al. (29) was synthesized by ONTORES Biotechnologies (Zhejiang, China). The identity and purity of the peptide was analyzed by analytical reversed-phase high-performance liquid chromatography (RP-HPLC) and mass spectrometry MALDI-TOF (purity >95%). Class-B CpG-ODN 1826 (5'-TCCATGACGTTCCTGACGTT-3') with total phosphorothioate-modification was provided by Operon Technologies, Alameda, CA, United States. To prepare Coa-ASC16-based formulations, FhKTM peptide and/or CpG-ODN were added to a dispersion of 2% (w/v) ASC16 in 5% dextrose solution, heated up to 72°C for 15 min, and then allowed to reach room temperature as described previously (27).

### Vaccination With FhKTM/CpG-ODN/Coa-ASC16

BALB/c mice were randomly divided into four groups (*n* = 4–5) as described in Table 1. Immunizations were performed three times at one-week intervals over 2 weeks. Each mouse was subcutaneously immunized with an entire dose (250 μl) equally distributed at five sites: tail, back, neck region, and both hind limbs (50 μl/site). CpG-ODN was administered at 75 μg/mouse/dose. The FhKTM dose was 10 μg/mouse/dose. One week after the last immunization, all groups were orally infected with 6 metacercariae of *F. hepatica* (Sanabria Laboratory, Universidad Nacional de la Plata, La Plata, Argentina). Mice were sacrificed at three different days, 0, 4, and 24, after infection.

**Table 1:** Processing mice in each group.

**Groups**	**Treatments**
Untreated	Non-immunized and uninfected
Infected	Non-immunized and infected
CpG-ODN/Coa-ASC16	Immunized with adjuvant and infected
FhKTM/CpG-ODN/Coa-ASC16	Immunized with FhKTM/CpG-ODN/Coa-ASC16 and infected

### Cytokine Detection Assay

Peyer patches (PPs) were harvested from the small intestine of mice and then incubated in RPMI 1640 medium (Gibco BRL, Life Technologies, Grand Island, NY) containing 0.5 mg/ml collagenase, 2% (V/V) fetal bovine serum (FBS; Thermo Fisher Scientific), 100 U/ml penicillin, and 100 μg/ml streptomycin for 30 min. The PPs cells were filtered through a cell strainer (100 μm; BD) and washed with the medium without collagenase. The cells were suspended in RPMI 1640 medium containing 10% (V/V) FBS, 55 μM 2-mercaptoethanol, 100 U/ml penicillin, and 100 μg/ml streptomycin and then cultured at 1.0 × 10^5^ cells/well in a U-bottom 96-well plate stimulated with FhKTM (2 μg/well) for 3 days at 37°C under 5% of CO_2_ and 95% air. Spleen, mesenteric lymph nodes (MLNs), and inguinal lymph node (ILNs) cells were obtained, homogenized, and suspended in RPMI 1640 medium (Gibco BRL, Life Technologies, Grand Island, NY) supplemented with 10% FCS (Gibco), 1 mM sodiumpyruvate, 2 mM l-glutamine, 100 U of penicillin/ml, and 100 μg/ml of streptomycin (complete medium). Cultures were incubated at 37°C in a humidified atmosphere (5% CO_2_) and stimulated with FhKTM (2 μg/ml) for 72 h. At the end of the incubations, cell culture supernatants were collected, aliquoted, and frozen at −80°C until being analyzed for IFN-γ, IL-17A, IL-4, IL-5, and IL-10 by sandwich ELISA according to the manufacturer's guidelines (BD Pharmingen, San Jose, CA, United States).

### Treatment With αIL-17A Antibody

Monoclonal antibody was applied to the vaccinated group to induce the functional inhibition of IL-17A. Two days before and after infection, FhKTM/CpG-ODN/Coa-ASC16 vaccinated and infected mice were injected with 250 μg (100 μl i.p./mouse/dose) of αIL-17A antibody (Invitrogen, Thermo Fisher Scientific, Waltham, MA, United States). The FhKTM/CpG-ODN/Coa-ASC16 and infected groups were alternatively injected with the non-specific isotype control IgG (Invitrogen,Thermo Fisher Scientific, Waltham, MA, United States) (100 μl i.p./mouse/dose) (30).

### Survival Curves

Mortality and survival of mice in different groups were observed until the completion of the experiment and survival curves were plotted until day 75 post-infection by using GraphPad Prism 6.01 software (GraphPad Software, San Diego, CA).

### Liver Analysis

The analysis of livers consisted of two parts. Gross lesions were scored (range 0 to 5) according to the method described by Changklungmoa N et al. (31) taking into account the extension of damage on the surface of livers_._ The histopathological examination was done after livers were fixed in 10 % neutral-buffered formalin for 48 h, followed by paraffin embedding. Sections of 5 μm were stained with hematoxylin and eosin (HE). Histological samples were scored according to Chien-Chang Chen et al. (32) with modifications. The lesions were scored between 0 and 9 based on the following findings: infiltration of inflammatory cells (score range, 0 to 3), together with the evaluation of liver tissue damage (necrosis, hemorrhagic foci, fibrosis, score range 0 to 3), and presence of tunnels and flukes (score range, 0 to 3), with 0 as normal and 9 as the most diseased.

### Antigen Specific Antibody Titers

FhKTM-specific titers of IgA in fecal extracts and IgG isotypes (IgG1 and IgG2a) in serum were determined by ELISA. Fecal extracts were prepared by suspending five fecal pellets in 0.5 ml of extraction buffer (100 μg/ml soybean trypsin inhibitor, Sigma Aldrich St. Louis, MO, United States), 10 mg/ml bovine serum albumin (Sigma Aldrich, St.Louis, MO, United States), and 30 mM disodium EDTA in PBS (pH=7.6). After homogenization and centrifugation at 4°C, the supernatants of the fecal extracts were used for IgA determination in feces (33). Blood was allowed to clot, and serum was removed and stored at −20°C until use. The small intestinal contents were flushed out with 3 mL of PBS. The intestinal lavage fluids were centrifuged at 9,200 g for 5 min at 4°C and the supernatants were stored at −80°C until analysis. For ELISA, FhKTM was diluted at 10 μg/ml in NaHCO3 1M (pH=9.6), and ELISA plates were coated in 100 μl/well overnight at 4°C. Plates were blocked with 5% bovine serum albumin (BSA) in PBS at 37°C for 1 h and washed with PBS-Tween 0.05%. Samples were incubated for 2 h at room temperature and after washing, rat anti-mouse IgA-HRP (BD Pharmingen, San Jose, CA, United States) or anti-mouse IgG-HRP (Invitrogen, Thermo Fisher Scientific, Waltham, MA, United States) diluted in 1% PBS–BSA were added for 1 h at room temperature. Finally, detection was performed with BD Opt EIA™ TMB Substrate Reagent Set (BD, San Diego, CA, United States). Titers were calculated as the reciprocal of the last serum dilution that yielded an absorbance at 490 nm above that of twice the mean value of blank. The sera from the non-immunized group (untreated) were represented by a full line.

### ALT and AST Measurement

The serum concentrations of alanine aminotransferase (ALT) and aspartate aminotransferase (AST) were determined using a kinetic-UV method by BIOCON laboratory, Cordoba, Argentina, under the established manufacturer's protocols.

### Statistical Analysis

Data were analyzed using GraphPad Prism 6.01 software (GraphPad Software, SanDiego, CA). Data analysis included one-way ANOVA followed by a Tukey's post-test for multiple comparisons and the unpaired Student's *t*-test. In survival experiments, Kaplan–Meier curves were analyzed with log-rank test. All data were considered statistically significant for *p*-values of * < 0.05, ** < 0.01 or *** < 0.001 depending on the experiment.

## Results

### FhKTM/CpG-ODN/Coa-ASC16 Vaccination Protects Against *F. hepatica* Infection

**Figure 1:**
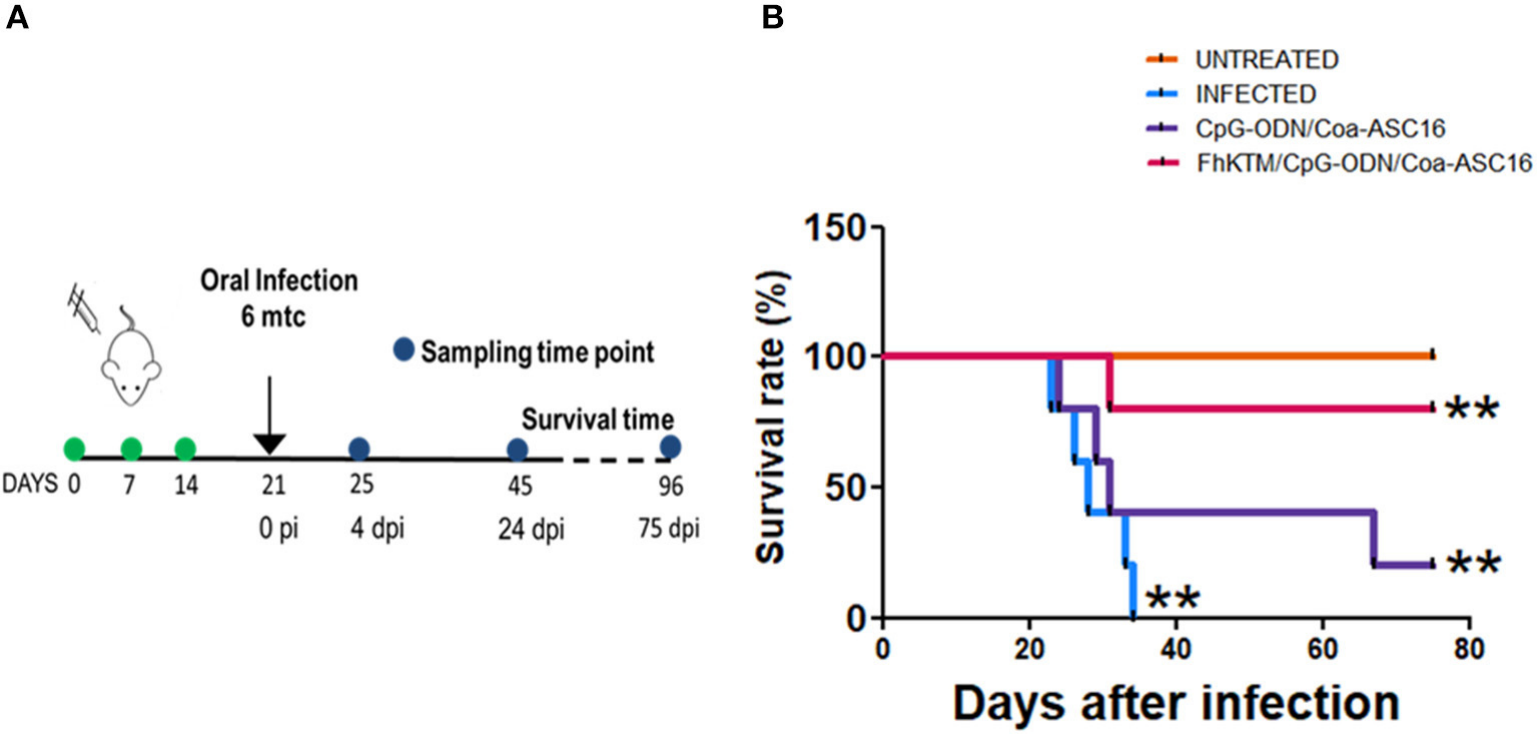
Vaccination with FhKTM/CpG-ODN/Coa-ASC16 increases the survival rate in *F. hepatica* infected mice. **(A)** Scheme of the vaccination and challenge experiments. BALB/c mice were injected s.c. in the upper and dorsal region on days 0, 7, and 14 with FhKTM/CpG-ODN/Coa-ASC16, CpG-ODN/Coa-ASC16, or PBS (infected). One week later, the mice were oral-challenged with 6 metacercariae of *F. hepatica*. Non-vaccinated non-infected (untreated) mice were used as a negative control group. The samples were obtained at 4 and 24 dpi. **(B)** Survival was monitored for >75 days. Each group comprised of five mice. Survival was significantly higher in the FhKTM/CpG-ODN/Coa-ASC16-immunized mice than in the control groups. Kaplan–Meier curves were generated and survival was compared across groups using the log-rank test **p < 0.05. This figure is representative of two experiments with similar results.

**Figure 2:**
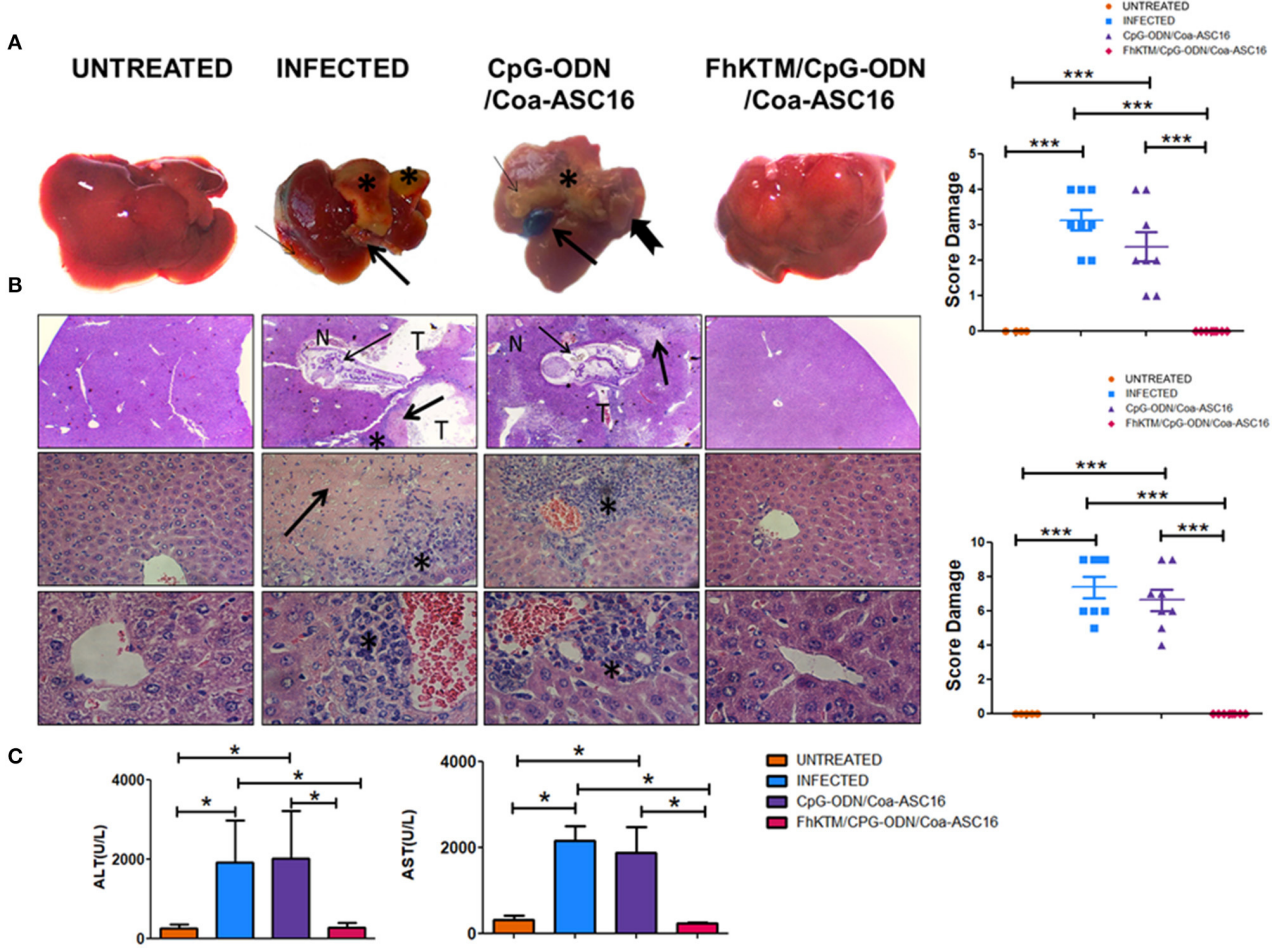
FhKTM/CpG-ODN/Coa-ASC16 immunization in mice prevented liver damage caused by *F. hepatica* infection. Gross lesions of livers from mice after 24 days of the infection with *F. hepatica* or without infection. **(A)** Untreated: tissue from mice without treatment showed a standard architecture with no observed lesions. Infected: there are marked irregularities of capsule (thin arrow), changes in the tissue color (asterix) belonging to the growing fibrosis generated, and enlargement of bile ducts (thick arrow). CpG-ODN/Coa-ASC16: multiple foci of fibrosis (asterix), also enlargement, thickening, and color changes due to hemorrhages in the gallbladder (thick arrow) and the presence of worms on the liver surface (notched arrow). FhKTM/CpG-ODN/Coa-ASC16: the livers maintained a structure similar to the untreated control mice without visible lesions. Right, the score was assessed by the extensión of liver damaged (score range 0 to 5). The data shown are pooled from two independent experiments with total (*n* = 5–8 per group). **(B)** Histopatological evaluation of livers from the different groups was performed 24 days after infection. Left, untreated control mice showed the expected mice hepatic architecture (left panel) [H/E, 100 × (top) 400× (middle panel) 900× (below)]. The infected group exhibited migrating newly excysted juvenile fluke (NEJ) in the hepatic parenchyma (thin arrow), associated tract (T), extensive Inflammatory infiltrate (*), moderate hepatocyte necrosis (N), and fibrotic connective tissue proliferation (thick arrow) at the chronic stage of the infection (center left panel). Likewise, CpG-ODN/Coa-ASC16 control evidenced the presence of NEJ and the consequences thereof (center right). FhKTM/CpG-ODN/Coa-ASC16 mice maintained the liver parenchyma structure despite infection (right panel). Right, histological samples were scored between 0 and 9 based on the following findings: infiltration of inflammatory cells (score range, 0 to 3), together with the evaluation of liver tissue damage (necrosis, haemorragic foci, fibrosis, score range 0 to 3), and the presence of tunnels and flukes (score range, 0 to 3), with 0 as normal and 9 as the most diseased. The data shown are pooled from two independent experiments with total (*n* = 5–8 per group). **(C)** Serum was collected at the time of euthanasia, 24 days after challenge. The levels of AST (right), aspartate aminotransferase; ALT (left), and alanine aminotransferase were determined by using a kinetic-UV method, under the established manufacturer's protocols. Data were analyzed by one-way ANOVA and Tukey's post-test *P < 0.05; ***P < 0.001. Data are shown as mean ± SD. All data are representative of two individual experiments.

To study whether FhKTM/CpG-ODN/Coa-ASC16 protects against *F. hepatica* infection, we followed an experimental procedure of immunization and infection described in Figure 1A. Samples from mice were obtained on days 25 and 45 after the first immunization (Figure 1A). In addition, the survival rates of infected mice were evaluated until 75 days post infection (dpi). Figure 1B shows that all infected mice died by day 32 pi, while immunization with FhKTM/CpG-ODN/Coa-ASC16 effectively increased mice survival, showing no significant differences with untreated animals. Moreover, mice injected only with CpG-ODN/Coa-ASC16 showed a survival impairment, with their survival significantly lower than it was observed for FhKTM/CpG-ODN/Coa-ASC16-vaccinated mice (Figure 1B). Taking into account that vaccination with FhKTM/CpG-ODN/Coa-ASC16 prolonged infected mice survival, we investigated the level of damage in the liver, the target organ of infection, establishing a macroscopical score (range 0 to 5) according to the extension of surface liver lesions. The infected and CpG-ODN/Coa-ASC16-injected mice showed significantly higher scores of liver lesions than the FhKTM/CpG-ODN/Coa-ASC16 vaccinated group, which did not present damage in the liver (Figure 2A). All vaccinated mice exhibited a microscopically preserved liver architecture comparable to the untreated group (Figure 2B). In contrast, livers from both infected and CpG-ODN/Coa-ASC16-injected mice presented migratory tunnels (T) containing young flukes (thin arrows), large areas of fibrosis that replace hepatic parenchyma (thick arrows), and large leukocyte infiltrates (asterisk) (Figure 2B). Results showing the histopathological analysis of the livers are summarized in Figure 2B. Accordingly, FhKTM/CpG-ODN/Coa-ASC16-immunized mice showed serum ALT and AST levels similar to those observed in untreated animals (Figure 2C). As expected, ALT and AST levels were significantly increased in sera from infected and CpG-ODN/Coa-ASC16-injected mice (Figure 2C). In summary, high survival rates and no significant changes in the liver structure, together with normal concentrations of hepatic enzymes (ALT and AST), demonstrate the effectiveness of the FhKTM/CpG-ODN/Coa-ASC16 vaccine to protect mice against *F. hepatica* infection.

### FhKTM/CpG-ODN/Coa-ASC16 Immunization Elicits Strong Antigen-Specific Humoral Immune Responses

**Figure 3:**
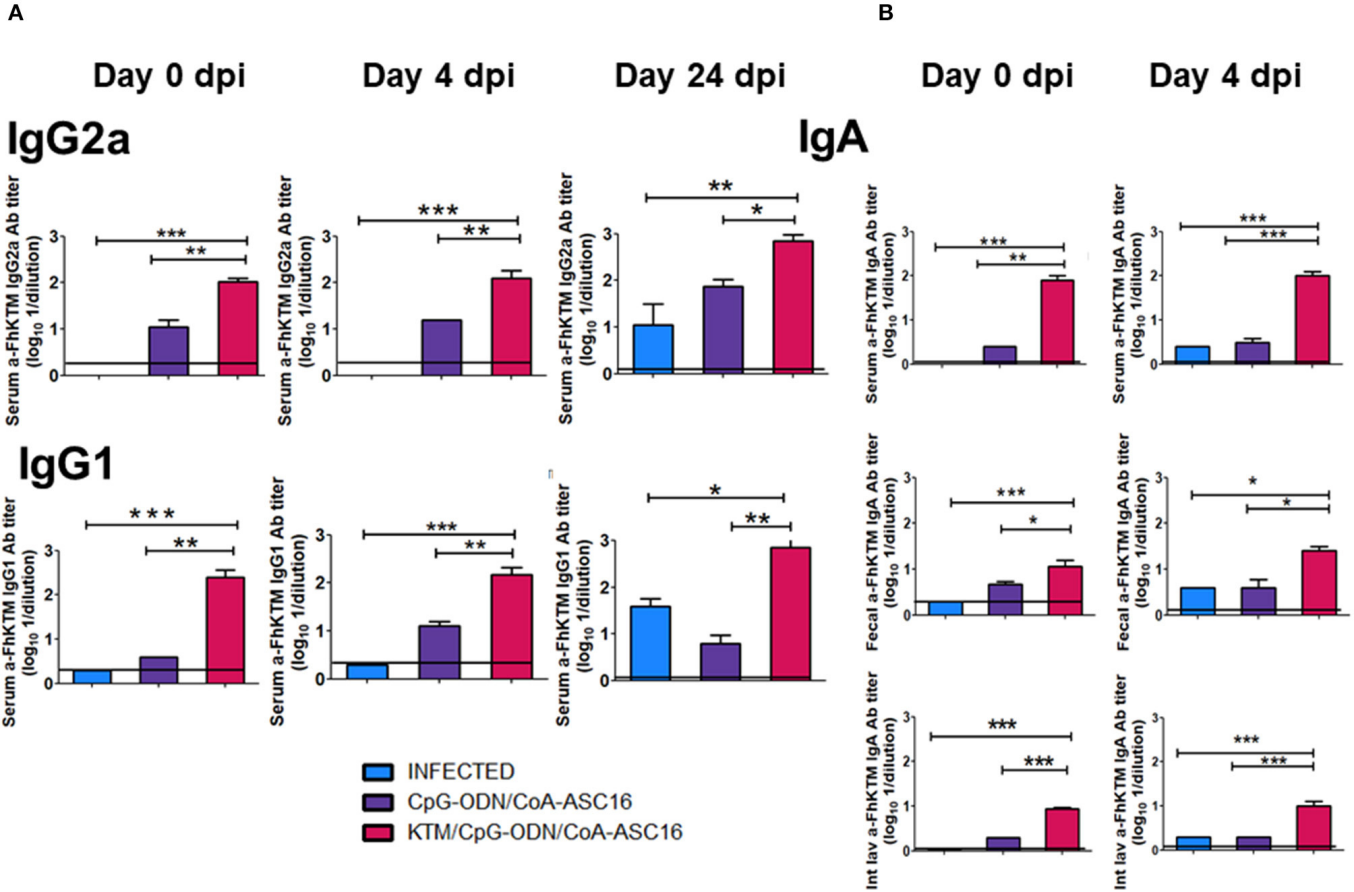
FhKTM-specific IgG antibodies detection. **(A)** IgG1 and IgG2a subclass antibodies in the sera of the immunized mice by enzyme-linked immunosorbent assay (ELISA). Serum samples were collected from the mice by retro-orbital bleeding at 0, 4, and 24 dpi. Humoral immune responses were analyzed. **(B)** IgA titles were detected in the acute infection on days 0 and 4 in sera, feces, and intestinal lavage. Results have been expressed as the mean of OD 450 ± SD values (*n* = 15) and are representative of at least three independent experiments. The data were analyzed by one-way ANOVA and Tukey's multiple comparison tests*p < 0.05; **p < 0.01; ***p < 0.001.

It has been reported that one important protective mechanism against *F. hepatica* is the humoral response (14, 18, 34, 35). Therefore, to evaluate whether FhKTM/CpG-ODN/Coa-ASC16 promotes an antigen-specific antibody response and primes the infection-induced humoral response, the levels of IgG1 and IgG2a antibodies against FhKTM were determined by ELISA in sera from FhKTM/CpG-ODN/Coa-ASC16-immunized, infected, and CpG-ODN/Coa-ASC16-injected mice on 0, 4, and 24 dpi. The anti-FhKTM IgG2a and IgG1 titers are depicted in Figure 3A, with the levels of anti-FhKTM obtained in untreated mice indicated as a line. Seven days after the third immunization (0 dpi, Figure 1A), FhKTM/CpG-ODN/Coa-ASC16-immunized mice showed significantly higher titers of FhKTM-specific IgG1 and IgG2a antibodies than CpG-ODN/Coa-ASC16-injected mice (Figure 3A). In addition, immunization worked as an effective stimulus for boosting the infection-induced antibody response, because at 4 and 24 dpi, FhKTM/CpG-ODN/Coa-ASC16-immunized mice showed significantly higher titers of IgG1 and IgG2a anti-FhKTM than those observed in serum from the other two infected experimental groups (infected and CpG-ODN/Coa-ASC16) (Figure 3A). Next, to evaluate the IgA immune responses induced by the vaccine formulation systemically and at a mucosal level, titers of FhKTM-specific IgA in serum, fecal pellets, and intestinal lavage were determined by ELISA (Figure 3B). According to what was observed in the systemic response for IgG1 and IgG2a, strong IgA responses were observed by vaccination with FhKTM/CpG-ODN/Coa-ASC16. Together, these data indicate that this vaccine is effective at inducing a specific antibody response at a systemic level and also in the intestine, with the latter being really important considering the migration period of the parasite through the host intestine wall at an early time after infection.

### Immunization With FhKTM/CpG-ODN/Coa-ASC16 Enhances Antigen-Specific IL-17A and IFN-γ Production

**Figure 4:**
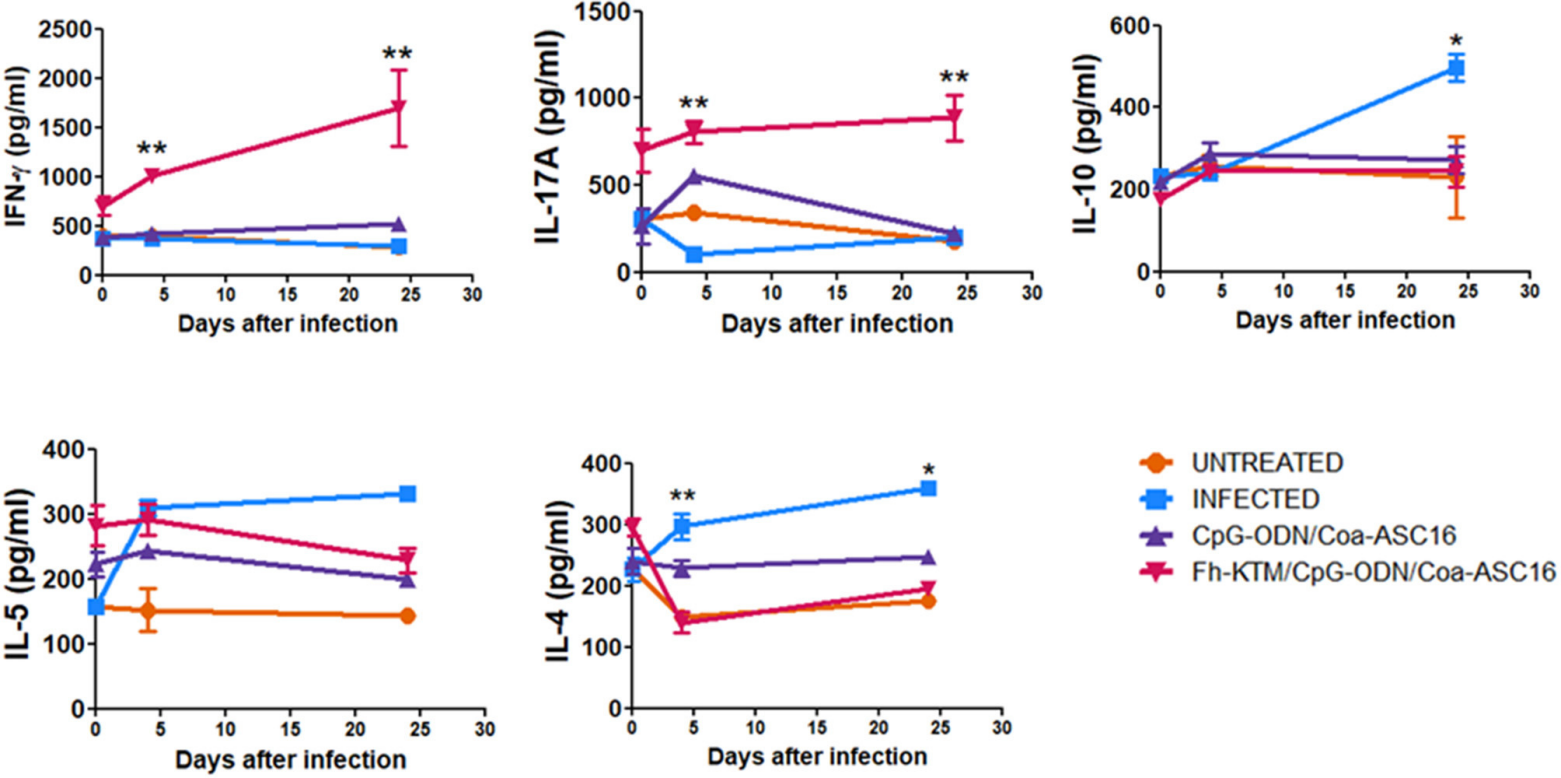
FhKTM-specific cytokine immune responses in BALB/c mice after vaccination. At 0, 4, and 24 dpi, spleens were collected and splenocytes were stimulated *in vitro* with FhKTM for 72 h. The culture supernatants from spleen cells were assessed for the production of IL-17A, IFN-γ, IL-10, IL-5, and IL-4 by ELISA. Results are shown as mean ± SD and levels of significance as indicated by *p*-values and are representative of two or three independent experiments. They were assessed by one-way ANOVA and Tukey's multiple comparison tests *p < 0.05; **p < 0.01.

**Figure 5:**
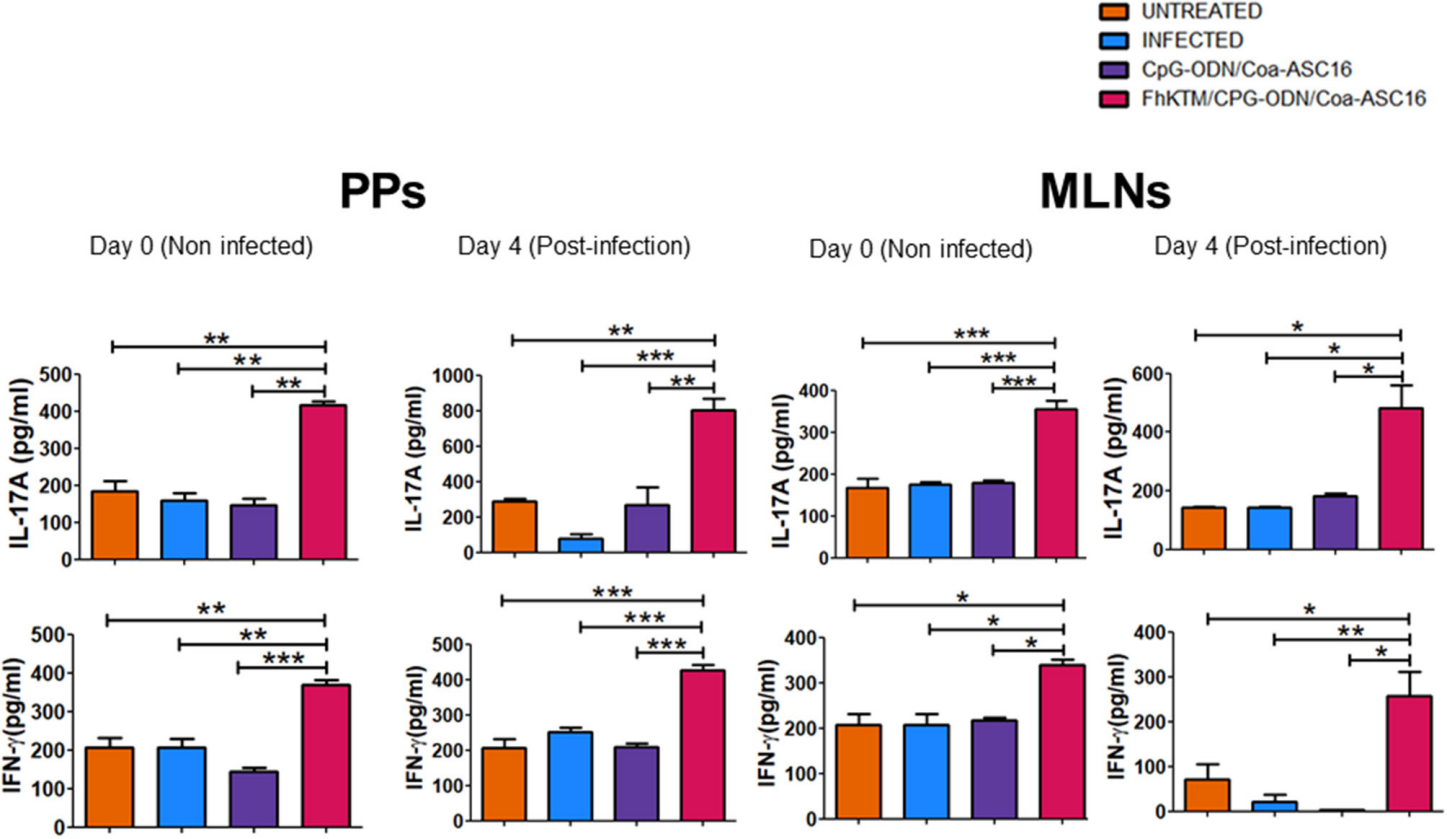
KTM/CpG-ODN/Coa-ASC immunized mice showed increased production of IFN-γ and IL-17A in supernatants from PPs and MLNs. Cell suspensions from PPs and MLNs from treated mice at day 0 (uninfected) and 4 dpi were cultured for 3 days with FhKTM. The IFN-γ and IL-17A were measured in the culture supernatants by ELISA test. Results are shown as mean ± SD and levels of significance as indicated by *p*-values, were assessed by one-way ANOVA and Tukey's multiple comparison tests *p < 0.05; **p < 0.01; ***p < 0.001.

Next, we evaluated whether vaccination with FhKTM/CpG-ODN/Coa-ASC16 is also able to promote an antigen-specific cellular response. To this end, BALB/c mice that were vaccinated and infected according to the scheme of Figure 1A were euthanized on 0, 4, and 24 dpi and the capacity of splenocytes to produce IFN-γ, IL-17A, IL-10, IL-5, and IL-4 after restimulation with FhKTM was assessed in the culture supernatants by ELISA. As shown in Figure 4, vaccination with FhKTM/CpG-ODN/Coa-ASC16 was able to induce a strong cellular response characterized by enhanced secretion of IL-17A and IFN-γ, whereas only a weak production of IL-4 and IL-5 was observed, thereby suggesting the stimulation of dominant Th17 and Th1 responses. Moreover, the IL-17A and IFN-γ production was markedly increased after the infection (Figure 4). As previously described (36), *F. hepatica* infection induces an increase in IL-4-, IL-5-, and IL-10-producing splenocytes, while FhKTM/CpG-ODN/Coa-ASC16-immunized mice not only did not increase IL-10- production but also decreased IL-4-producing splenocytes after the infection (Figure 4). Taking into account that an early IL-17A production has been previously demonstrated to promote IgA class switching in lymph organs (37), and considering our data showing increased IgA in feces as well as in intestinal lavage, we examined cytokine production by lymphatic organs for mucosal immunity, such as MLNs and PPs. Figure 5 shows that after three immunizations and prior to infection, MLNs or PP cells from FhKTM/CpG-ODN/Coa-ASC16-immunized mice produced high levels of IL-17A and IFN-γ after antigen-specific stimulation. In addition, MLNs or PP cells from vaccinated mice secreted higher levels of these cytokines compared to those secreted by cells of MLNs or PP from CpG-ODN/Coa-ASC16 and infected mice (Figure 5).

These data suggest that IL-17A and IFN-γ production could generate an inflammatory environment during the parasite migration which might contribute to its elimination.

### *In vivo* Neutralization of IL-17A Abolishes the Protective Capacity of FhKTM/CpG-ODN/Coa-ASC16 Vaccination

**Figure 6:**
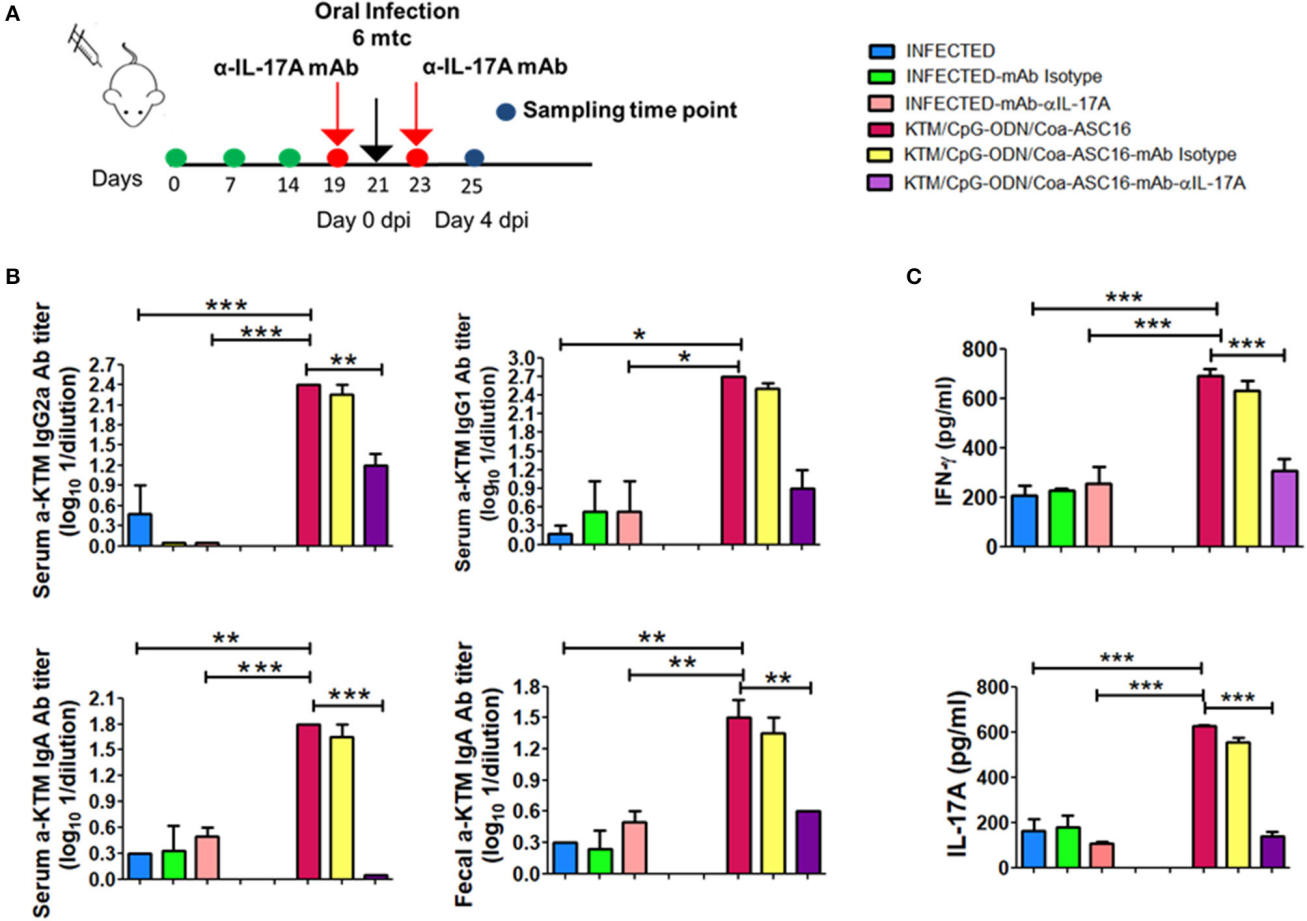
IL-17A neutralizing reduced the immune response against FhKTM inFhKTM/CpG-ODN/Coa-ASC16 immunized mice. **(A)** Mice were administered with anti-IL-17A antibody and the corresponding isotype control (250 μg i.p) 2 days before and after infections. **(B)** Twenty-five days after the first immunization, serum and feces samples were obtained and FhKTM antibody levels were measured by ELISA. Their titers were detected by serial serum dilution and the limit was determined by twice the value of the blanks mean. **(C)** Both IL-17A and IFN-γ secretion were evaluated in the splenocytes supernatant culture by ELISA. dpi: days post infection. Data are representative of two independent experiments. One-way ANOVA + Tukey'multiple comparison tests. *p < 0.05, **p < 0.01, ***p < 0.001.

**Figure 7:**
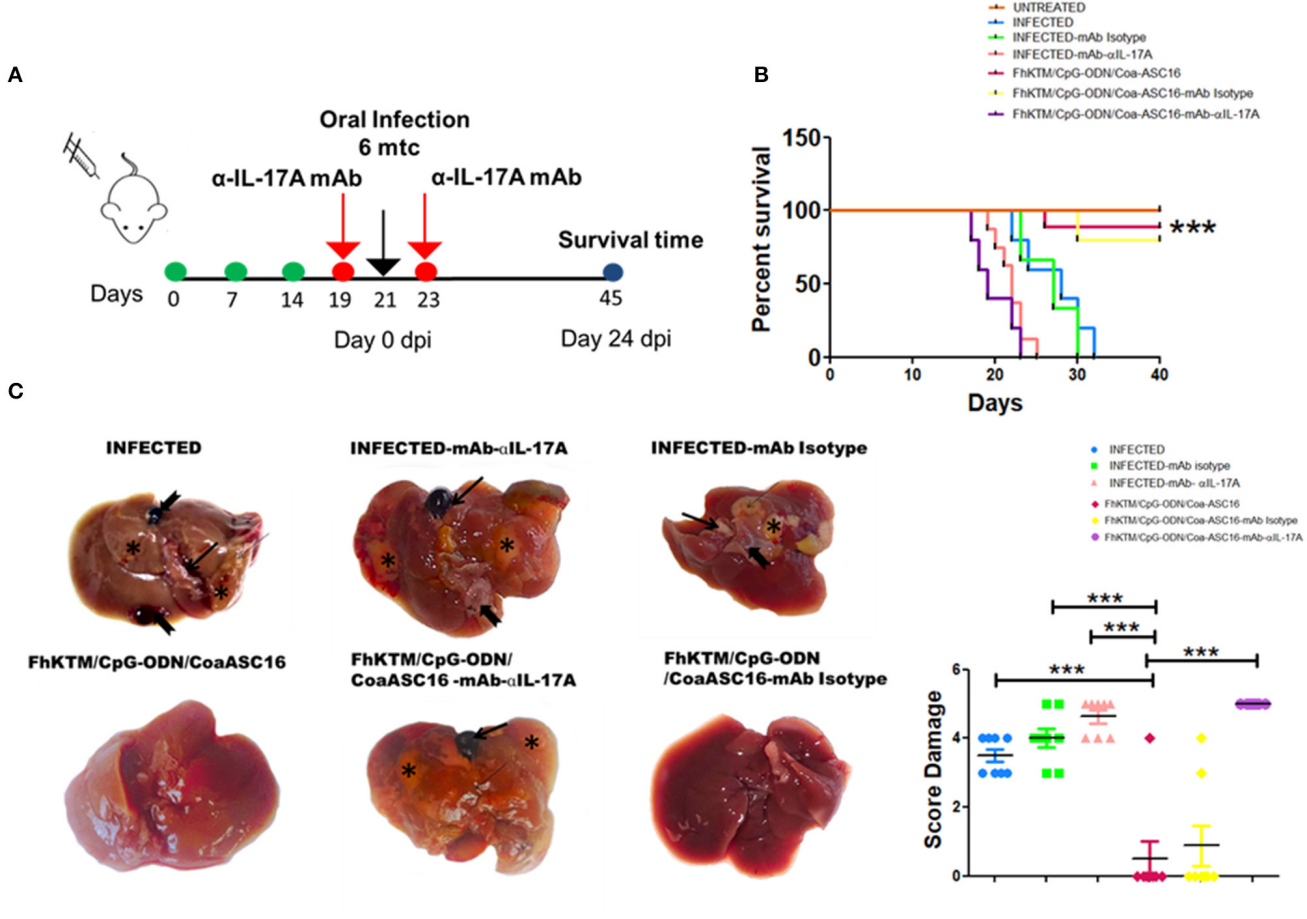
IL-17A neutralization decreased the efficacy of FhKTM/CpG-ODN/Coa-ASC16 vaccination. **(A)** Mice were administered with anti-IL-17A antibody and the corresponding isotype control (250 μg i.p) 2 days before and after infections. **(B)** Kaplan–Meier graphs show survival curves for those mice immunized three times with FhKTM/CpG-ODN/Coa-ASC16 or PBS. IL-17A-mAb and corresponding isotype control (mAb Isotype) were administered 2 days both before and after infection. dpi: days post infection. **(C)** Left: infected liver showing marked enlargement with distended gallbladder (thick arrow), areas of fibrosis extending (asterix), and larvae on the parenchyma surface (notched arrow). FhKTM/CpG-ODN/Coa-ASC16: liver showing normal size with no observed lesion except in 1 out of 8 mice. Infected-mAb-IL-17A: the liver showing marked severe macroscopic damage (thin arrow) and presented irregular surface denoting hepatic fibrosis (asterix). The presence of larvae on the surface can be seen (notched arrow). FhKTM/CpG-ODN/Coa-ASC16 -mAb-IL-17A: The hepatic parenchyma is severely reduced, firm, and presents an irregular surface (thin arrow) denoting hepatic fibrosis (asterix). Abnormal gross appearance of the distended gallbladder with presence of blood inside (thick arrow). infected-mAb Isotype: the liver is smaller than normal and presents irregular surface tissue denoting hypertrophied (thin arrow) and replaced with fibrosis (asterix) and a fluke released after the rupture of the gallbladder (notched arrow). FhKTM/CpG-ODN/Coa-ASC16-mAb Isotype: liver showing no apparent gross pathology except for in 2 out of 8 mice. Right, the score damage was assessed by the extensión of liver damaged (score range 0 to 5). The data shown are pooled from two independent experiments with total (*n* = 5–8 per group). The statistical differences among survival curves were calculated by using Mantel–Cox test. ***p < 0.001.

IL-17 production has been associated with plasma cells switching to IgG2a antibodies and the promotion to IgA isotype (38, 39). In addition, IFN-γ has been involved in the protection against *F. hepatica* (19, 20, 40). Moreover IL-17A can act synergistically with IFN-γ to activate antiparasitic mechanisms by macrophages (41). Taking into account these reports and our results showing high levels of IL-17A after vaccination, we decided to investigate the role of IL-17A on vaccine-induced protection. Groups of FhKTM/CpG-ODN/Coa-ASC16-immunized or PBS-treated (infected) mice received neutralizing IL-17AmAb or isotype-matched control mAb 2 days before and after the oral challenge with the metacercariae (Figure 6A). Injection of neutralizing α-IL-17A mAb, but not control mAb, significantly decreased the serum levels of vaccine-induced IgG2a and IgA as well as fecal IgA titles, but did not significantly affect IgG1 production (Figure 6B). Likewise, splenocytes from vaccinated mice that were treated with α-IL-17A mAb, but not control mAb, showed diminished IFN-γ production after antigen stimulation in culture (Figure 6C). In addition, the vaccine-induced protection appeared to be mediated by an IL-17A-driven immune response because treatment of mice with neutralizing α-IL-17A mAb abolished the protective effect evaluated as survival rate (Figures 7A,B). Thus, FhKTM/CpG-ODN/Coa-ASC16-immunized mice and those treated with anti-IL17A neutralizing antibody decreased their survival compared with vaccinated animals without treatment (lines violet vs. fucsia) (Figure 7B) and increased the gross liver damage (Figure 7C). Interestingly, the IL-17A neutralization decreased the survival of *F. hepatica* infected mice with or without treatment (lines pink and light blue), suggesting an important role of IL-17A in the protection against *F. hepatica*. These data highlight the remarkable role played by IL-17A as a regulator of IFN-γ production and specific antibody response which correlates with the survival levels found in mice after the infectious challenge.

## Discussion

The production of an efficient vaccine against *F. hepatica* remains a major challenge for the scientific community for different reasons. The anti-helminthic resistance, high rates of reinfection in endemic areas, and the acute infection cases provoking animal death have raised the need for developing a vaccine against fasciolosis (1, 14). However, the most important challenge in vaccine design against this helminth is the possibility of beating the Th2-type or immunosuppressive responses with a view to an efficient response to eliminate the parasite. So far, numerous vaccination attempts have included different purified parasite molecules or their recombinant forms in cattle, sheep, and goats with varying levels of protection (14, 42–44).

To date, the vaccination trials developed against *F. hepatica* are not reproducible among animal models, in which variable levels of protection are achieved regardless if the antigen is native or recombinant. A number of antigens have been tested as promising vaccine candidates in mice (22, 45, 46). However, partial protection, insufficient improvement in animal survival, or hepatic damage is not be enough to merit progress in the development of a commercially viable vaccine for livestock production. For these reasons, it is still important to define new vaccine candidates and efficient adjuvant formulations in murine models prior to the examination of the protective capacity in natural hosts such as cows or sheep.

The rationale for the vaccine design in this work was based on the properties of FhKTM. Apart from being an abundant protein within the parasite gut, the parenchymal tissue, and the tegument of juvenile (NEJ) (25) and adult (29), its role as protease inhibitor enables the parasite to avoid both its own and the host's deleterious protease action. Along with this, the antigen was formulated with a novel adjuvant strategy, CpG-ODN/Coa-ASC16, that constitutes a nanoplatform. This adjuvant strategy is able to induce potent Th1 and Th17 responses, and elicit long-term antibody responses (27, 28). In this study we demonstrated that the immunization of mice with FhKTM/CpG-ODN/Coa-ASC16 increases the survival against *F. hepatica* challenge. Accordingly, the immunized animals presented a highly preserved liver structure, suggesting that mice vaccination somehow prevented worms from reaching the liver. This idea is also supported by results showing that immunization induces early production of specific antibodies and cytokines associated with INF-γ and IL-I7 protection, both systemically and locally in MLNs and PPs after infection. On the one hand, high levels of FhKTM-specific IgA in the intestinal content as well as in feces from vaccinated mice could favor the hypothetical expulsion of parasites. Although *F. hepatica* is a trematode that remains for a short period of time in the intestine, the mechanisms of parasite expulsion in the gut as a result of vaccination could also be operating against the larval stage of this parasite. As described by others, the transference of IgA or IgG1 antibodies from resistant mice to helminth infections confers partial resistance to different nematodes (47, 48), probably through their neutralizing effect on secreted parasite antigens, or by trapping larvae (49–51). In addition, we cannot not rule out the possibility that antibodies generated during immunization with the vaccine might participate in mechanisms of antibody-dependent cell-mediated cytotoxicity (ADCC) and reactive oxygen and nitrogen species (ROS and NOS), according to results reported in *in vitro* studies by Piedrafita et al. (52). On the other hand, a critical role for IL-17A in the protective immunity against *F. hepatica* shown in this study is an interesting finding, whereas the Th1 profile has been the response mostly associated with protective mechanisms (19, 20, 53). The fact that IL-17A was crucial to induce the IgA isotype in the fecal content of vaccinated mice correlates with the ability of Th17 cells shown by other authors to become precursors for the follicular helper T cells in PPs and to induce IgA class switching (54). This fact could be explained by the capacity of IL-17A to increase the transport and secretion of IgA into the intestinal lumen (38). Moreover, Th17 cell-deficient mice had an impaired antigen-specific intestinal IgA after immunization with cholera toxin, pointing out that Th17 cells were responsible for inducing the switch of GC B cells toward the production of high-affinity T cell–dependent IgA (54). Given the important role of IL-17 in the protective immunity induced by the vaccine, we cannot rule out the presence of innate as well as adaptive cells as a possible source of Th17, since both CD4^+^ and CD4^−^IL17A-producing cells were found in the spleen of vaccinated animals (data not shown). Among the effector mechanisms of IL-17A there appears the ability to recruit neutrophils, which destroy the pathogen through the production of cytokines, chemokines, and anti-microbial peptides or myeloid cells which in turn restrict pathogen survival through activation and recruitment of Th1 cells (55). In the present study, a low neutrophil recruitment at the peritoneal cavity was observed in all experimental mice without significant differences among the groups (data not shown). On the other hand, the passage of worms through the intestinal wall that could induce neutrophil recruitment is random and transitory, so its finding might be difficult. The uncoupled IL-17A-dependent effector mechanisms from the neutrophil response have already been demonstrated in barrier tissues, in mouse models oropharyngeal, or skin fungal infections where IL-17A provided immunity through anti-microbial peptide generation (56), independently of neutrophils (57). These data suggest that the mechanism by which IL-17A plays a crucial role in the protective immunity against *F. hepatica* might be independent from neutrophil recruitment, and is still to be determined.

In addition, high systemic production of IFN-γ and IgG2A levels in vaccinated animals is in agreement with previous reports showing the association of these responses with increased levels of protection against the parasite (34). A close relationship between IL-17A and IFN-γ production was demonstrated in this work since the blockade of IL-17A significantly decreased IFN-γ levels in splenocyte supernatants and, consequently, animal survival. The precise mechanism by which the production of IFN-γ is dependent on IL-17 is unknown, however, it could be speculated that after this cytokine is produced, responder cells such as epithelial or myeloid cells through IL-17R signaling might induce the recruitment of Th1 cells. These cells could secrete pro-inflammatory cytokines, chemokines, and anti-microbial peptides to restrict the pathogenesis of the disease (55). Although these mechanisms have been proposed in different bacterial (58, 59) or protozoal infections (60), we cannot rule out that IL-17A might play a similar role during *F. hepatica* infection.

Data from other authors support the idea of a synergistic effect between IFN-γ and IL-17A in protective mechanisms against different pathogens through the potentiation of NO production in macrophages (41, 61, 62). Although a mucosal response in the intestine after subcutaneous immunization with FhKTM/CpG-ODN/Coa-ASC16 might seem surprising, recent findings have shown that parenteral immunization can generate a potent IgA response in mucosal tissues (63, 64). This fact could be explained in two hypothetical ways: in one of them, the antigen is captured by APC in the injection site and then transported to mucosal-associated lymphoid tissues (MALT) for antigen presentation. In the other, the antigen can be presented peripherally to naive T cells and B cells which are in turn home to mucosal tissues. The high levels of IFN-γ and IL-17A observed in inguinal lymph nodes after immunization with FhKTM/CpG-ODN/Coa-ASC16 (data not shown) suggest that the APC carrying the antigen might spread to the draining peripheral lymph nodes, either prior to or simultaneously with the antigen presentation to lymphocytes in the MALT. One limitation in this type of approach to studying protective immunity is the difficulty to decide whether protection comes from mucosal or systemic immunity, suggesting that induction of mucosal immunity by parenteral injection is an important issue for vaccine design. Finally, we believe that the CpG-ODN/Coa-ASC16 platform might allow FhKTM-long term release. Coa-ASC16 nanostructure have a certain rigidity, which can either modulate the release of molecule/s into the biological medium or provide stability to loaded molecules (65). Previously, it has been reported by *in vitro* approaches that Coa-ASC16 generates a sustained release of both OVA and CpG-ODN (27). In addition, Coa-ASC16 could exert a protective effect, avoiding FhKTM antigen protease degradation. This strategy may work *in vivo* as a depot effect, which often makes it possible to reduce the dose and/or the number of immunizations required for an optimal response.

The precise protective immunity mechanism as induced by FhKTM remains to be investigated. However, our data highlight the importance of designing vaccines that induce a potent response in mucosa and systemic levels capable of preventing the parasite from reaching the liver. Given the high levels of protection shown in mice susceptible to the infection, our next step is the validation of this vaccine system to the natural hosts of the infection, such as sheep, upon which our regional livestock economy is based.

## Data Availability Statement

The raw data supporting the conclusions of this article will be made available by the authors, without undue reservation.

## Author Contributions

LC and LS conceived of and designed the experiments and wrote the paper. LS, DC, PR, and MS performed the experiments. LC, LS, PR, LSC, CM, and BM analyzed the data. BM, SP, and DA conceived, developed, and tested the adjuvant capacity of CpG-ODN/Coa-ASC16. RS and CP produced the metacercariae. LC, CM, LSC, and PR contributed reagents, materials, and analysis tools. PR, LSC, BM, and CM revised the manuscript. All authors contributed to the article and approved the submitted version.

## Conflict of Interest

The authors declare that the research was conducted in the absence of any commercial or financial relationships that could be construed as a potential conflict of interest.
